# 

**Published:** 1889-11

**Authors:** 


PAGE.
Looking Forward..................241
The Absolute Tests of Death.....244
How to Live Long..............247
Our Daughters.................249
Practical Aids to beauty........250
Human Spontaneous Combustion.250
Music and Lectures..............251
Meats and their Digestibility....251
A New Preservative Process.....252
Why do People Marry?..........253
Effects of Stimulants of the Voice.254
What are Tyrotoxicon and Ptomains?....................255
Infant Feeding....................258
Natures Transformations.........258
Olive Oil in Scarlet Fever........259
Beans............................259
Weak Hearts.....................260
Household........................261
Miscellaneous....................262
Literary..........................264

INDEX TO ADVERTISEMENTS OF HALF A PAGE AND OVER.

PAGE.
Consumers Supply Association I
Celluloid...................... II
Hollow Suppositories......... III
E. C. Morris & Co............ IV
Lactopeptine.................. V
Christ Before Pilate.......... VI
Dennin's Certain Cure........ VII
An Unprecedented Offer...... VIII
The Herb Cure Co .......... X
Revised Premium List........ XI

